# Effect of transcranial direct current stimulation associated with hypocaloric diet on weight loss and metabolic profile in overweight or obesity: study protocol for a double-blind, randomized controlled clinical trial

**DOI:** 10.1186/s13063-018-2776-3

**Published:** 2018-07-16

**Authors:** Carina de Araujo, Raquel Crespo Fitz, Daniela Albugeri Nogara, Pedro Schestatsky, Fernando Gerchman

**Affiliations:** 1 Postgraduate Program in Medical Science: Endocrinology, Universidade Federal do Rio Grande do Sul, Porto Alegre, Rio Grande do Sul Brazil; 2 Endocrine Division, Hospital de Clinicas de Porto Alegre, Universidade Federal do Rio Grande do Sul, Rua Ramiro Barcelos 2350, Anexo, 4° andar, Porto Alegre, Rio Grande do Sul CEP: 90035-003 Brazil; 3 Medicine Graduate Course, School of Medicine, Universidade Federal do Rio Grande do Sul, Porto Alegre, RS Brazil; 4 Neurology Service, Hospital de Clinicas de Porto Alegre, Universidade Federal do Rio Grande do Sul, Porto Alegre, Rio Grande do Sul Brazil

**Keywords:** Clinical trial, Transcranial direct current stimulation, Neuromodulation, Weight loss, Obesity, Hypocaloric diet

## Abstract

**Background:**

Dietary interventions have limited success in promoting sustainable weight loss; new treatments allowing better compliance with hypocaloric diets should be developed. The aim of this trial is to describe the effects of a protocol combining repetitive active transcranial direct current stimulation (tDCS) with a hypocaloric diet on weight loss and food consumption in overweight or obese adults.

**Methods/design:**

Overweight or obese adults between 20 and 50 years of age with stable weight over the last 4 months will be selected for a 4-week randomized clinical trial of fixed-dose tDCS (20 sessions; 5 consecutive weekdays/wk, 2 mA, 20 minutes) over the right dorsolateral prefrontal cortex associated with a weight loss diet. The subjects will be randomly assigned in a 1:1 ratio and stratified by sex to active tDCS + diet or sham tDCS + diet. The study will be conducted at the Endocrine and Metabolism Unit of the Hospital de Clínicas de Porto Alegre, Brazil. The primary outcome is weight loss. Energy and macronutrient consumption, as well as adherence to the diet, will be assessed using 3-day weighed dietary records. Changes in blood glucose and plasma insulin will be assessed, and participants will complete self-report questionnaires to assess changes in mood and food behavior. All analyses will be done on a per-protocol and intention-to-treat basis.

**Discussion:**

This study explores the potential role of tDCS as an adjunctive treatment with a hypocaloric diet for obesity management.

**Trial registration:**

ClinicalTrials.gov, NCT02683902. Registered on 11 January 2016.

**Electronic supplementary material:**

The online version of this article (10.1186/s13063-018-2776-3) contains supplementary material, which is available to authorized users.

## Background

Obesity is a complex and multifactorial condition characterized by adipose tissue accumulation that is strongly associated with multiple comorbidities, including type 2 diabetes, cancer, cardiovascular diseases, sleep apnea, and physical and social limitations [1, 2]. Increased consumption of industrialized, processed, highly palatable foods rich in fat and sugar is being identified as a major contributor to the worldwide obesity epidemic [3]. As a consequence, limiting the consumption of foods with high caloric density could have an important impact in preventing and treating obesity [3].

Calorie-restricted diets associated with lifestyle intervention are the first-choice treatment for weight loss [4]. However, weight loss through hypocaloric diets could induce neuroendocrine changes that trigger an intense, subconscious, and powerful urge to eat, known as food craving [5]. The behavioral drive to find and eat food is closely related to the rewarding feeling that this food provides, resulting in a strong motivation to engage in this behavior again, which frequently results in failure to maintain a hypocaloric diet [5, 6].

Transcranial direct current stimulation (tDCS) is a noninvasive neuromodulation technique that provides a safe, painless, inexpensive, and nonrestrictive method to induce neuroplasticity [7, 8]. It has previously been shown that dorsolateral prefrontal cortex (DLPFC) neuromodulation controls the desire to eat and thus may be involved in food intake regulation. The use of tDCS has been studied as a promising treatment for food craving [9–11]. Moreover, two recent studies have demonstrated caloric intake reductions of approximately 32% after a single tDCS session [12] and 14.2% after eight consecutive tDCS sessions compared with controls [13]. This effect was related to reduced food intake, particularly carbohydrates [13].

Previous studies have demonstrated that repetitive tDCS sessions may have potential as a therapeutic intervention for decreasing the cravings associated with cocaine, alcohol, and smoking [14–16]. Because food and drug cravings share common biological mechanisms in the brain, repetitive tDCS sessions should yield similar results in overweight or obese individuals on a hypocaloric diet. However, despite the potential role of tDCS as a treatment for obesity, few studies have been conducted on overweight or obese individuals using this procedure [10, 17, 18]. To our knowledge, no study has been published evaluating a daily tDCS protocol as an adjuvant tool for weight loss during a hypocaloric diet.

In this context, we designed a randomized clinical trial to evaluate the effect of repetitive tDCS associated with a hypocaloric diet on food consumption and weight loss in overweight or obese adults. We hypothesize that subjects undergoing daily active tDCS associated with a hypocaloric diet would have better adherence to the prescribed diet than those undergoing sham tDCS, resulting in greater weight loss and an improved metabolic profile.

## Methods/design

### Design overview

Subjects in this 4-week double-blind, randomized, single-center, placebo-controlled trial will receive one of two interventions: (1) daily sessions of active tDCS + hypocaloric diet or (2) daily sessions of sham tDCS + hypocaloric diet.

After screening and selection visits, included participants will undergo a complete baseline assessment that includes a clinical and nutritional interview, as well as screening questionnaires to assess eating behavior and psychiatric disorders such as depression and anxiety. The participants also will undergo a standard protocol including a physical examination, anthropometric assessment, and metabolic and biochemical laboratory measurements. They also will receive individual counseling to improve dietary habits and will be prescribed a low-calorie diet by a dietitian to reduce 3% of their initial body weight over a 4-week treatment. The subjects also will undergo one tDCS session each weekday (active or sham stimulation according to previous randomization) during the 4 weeks of treatment (i.e., a total of 20 sessions). During these sessions, the subjects will also be exposed to a series of images of high-calorie foods known to stimulate the DLPFC and the appetite [19]. A subgroup of participants will use the FreeStyle® Libre™ Flash Glucose Monitoring System (Abbott Diabetes Care Inc., Alameda, CA, USA) during the intervention to assess whether this form of brain stimulation has any effects on glycemic variability. At the end of the 4-week intervention period, the participants will undergo the same baseline assessments, and their weight, body composition, and food-related behavior will then be reassessed after 3 and 6 months.

**Fig. 1:**
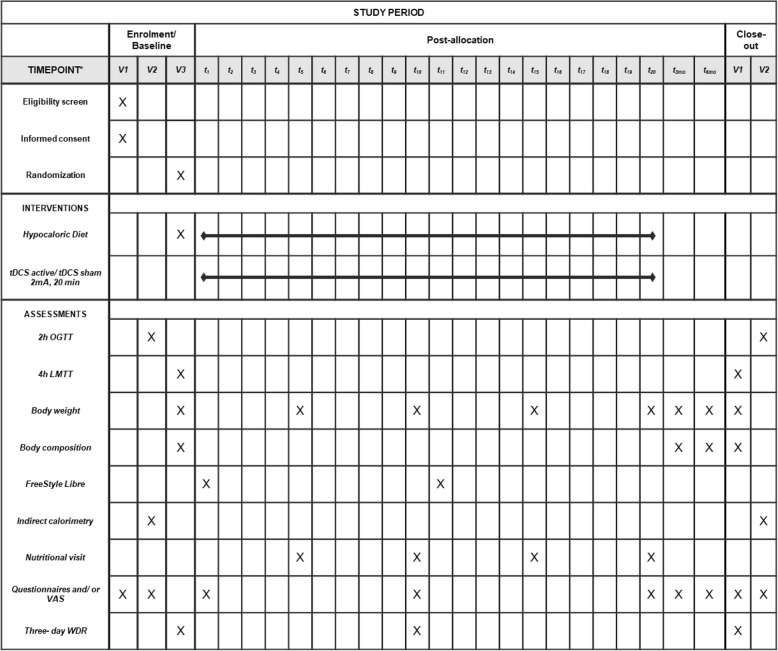
Standard Protocol Items: Recommendations for Interventional Trials (SPIRIT) diagram. *Time points: V1, V2, and V3 are visits 1, 2, and 3 in the respective periods (baseline or after 4 weeks of tDCS). t1 to t20 represent each tDCS/sham-tDCS session. t3mo and t6mo represent the evaluations performed 3 and 6 months after the end of the intervention period. *LMTT* Liquid meal tolerance test, *OGTT* Oral glucose tolerance test, *VAS* Visual analogue scale, *WDR* Weighed dietary records

The present protocol was written in accordance with Standard Protocol Items: Recommendations for Interventional Trials (SPIRIT) guidelines, with the SPIRIT checklist completed and construction of a flow diagram in order to optimize the quality of reporting [20] (Fig. 1 and Additional file 1).

### Participants

Overweight or obese subjects were recruited via advertisements on the Hospital de Clínicas de Porto Alegre website, in a local newspaper, and on television or through referral by physicians or nutritionists from clinics in the metropolitan area of Porto Alegre, a large city in southern Brazil. The study includes men and women with a body mass index (i.e., weight in kilograms divided by height in meters squared) between 25 and 35 kg/m^2^, aged 20–50 years, with stable body weight in the 12 weeks prior to screening, who had not received nutritional counseling within 6 months prior to screening, and who were able to comply with the requirements of the study protocol. Table 1 shows further details of the inclusion and exclusion criteria.

**Table 1:** Inclusion and exclusion criteria

Inclusion criteria	
• Adults of either sex, aged between 20 and 50 years	
• 25 ≤ BMI < 35 kg/m^2^ at screening	
• Stable weight for at least 12 weeks prior to screening	
Exclusion criteria	
• Women who are pregnant, breastfeeding, trying to become pregnant, or not using adequate contraception	
• Women in perimenopause, menopause, or postmenopause or who have had early menopause (under 40 years old) or a hysterectomy or oophorectomy	
• A history of severe cranial trauma with changes in cranial anatomy or metallic intracranial implants	
• Patients with preexisting irritations, cuts, or lesions where the tDCS electrodes would be placed	
• Type 1 diabetes; metabolic or acute complications of diabetes within the past 6 months	
• A history of any acute or chronic intestinal disease (e.g., Crohn’s disease, inflammatory bowel disease)	
• Having received nutritional counseling in the last 6 months by a nutritionist	
• A history of severe depression or other serious psychiatric comorbidities	
• A history of gastric bypass, antrectomy, or small bowel resection	
• A history of chronic pancreatitis or idiopathic acute pancreatitis	
• Myocardial infarction, coronary artery bypass surgery, posttransplant cardiomyopathy, or stroke in the last 6 months	
• Any abnormality in clinical laboratory tests that might prevent safe participation in the study	
• Diagnosed and/or treated tumor (except basal cell skin cancer, carcinoma in situ of the cervix, or prostate cancer in situ) in the last 5 years	
• A history of known hemoglobinopathy or chronic anemia	
• Blood donation of 1 U or more (≥ 500 ml) or significant blood loss (≥ 500 ml) in the last 2 weeks, or blood transfusion in the past 8 weeks	
• Oral antidiabetic drug treatment and/or herbal preparations or nonprescribed medications that could affect glycemic control in the last 12 weeks	
• Chronic oral or parenteral corticosteroid treatment (> 7 consecutive days) in the last 4 weeks	
• Weight loss treatment agents (e.g., orlistat, sibutramine, topiramate, bupropion) in the last 12 weeks	
• Ingestion of mineral oil or fiber supplements (e.g., Benefiber [GSK, Philadelphia, PA, USA], Metamucil [Procter & Gamble, Cincinnati, OH, USA])	
• Unstable doses of lipid-lowering drugs in the past 8 weeks	
• Unstable doses of thyroid hormone replacement in the past 12 weeks	
• Enrolled in another drug/device trial	
• Any laboratory abnormalities identified in screening, such as alanine aminotransferase (ALT) and/or aspartate aminotransferase (AST) more than three times the upper limit of normal, glomerular filtration rate estimated by the CKD-EPI equation ≤ 30 ml/min/1.73 m^2^, TSH outside the normal range, or triglycerides ≥ 400 mg/dl.	
• A history of active substance abuse (including alcohol) within the past year.	
• Any acute condition or exacerbation of a chronic condition that would, in the investigator‘s opinion, interfere with the procedures	

*Abbreviations: BMI* Body mass index, *CKD-EPI* Chronic Kidney Disease Epidemiology Collaboration, *tDCS* Transcranial direct current stimulation, *TSH* Thyroid-stimulating hormone

All those who responded to the advertisements (*n* = 322; 171 from the first campaign and 151 from the second) were contacted by telephone, and the study’s objectives were explained to them. Those interested in participating were assessed according to the inclusion and exclusion criteria. A total of 148 individuals were considered eligible and agreed to participate in a drawing. They were included in an Excel spreadsheet (Microsoft, Redmond, WA, USA) in alphabetical order and according to gender. Each participant was randomly assigned a value between 0 and 1 using the “=RANDOM()” function, and the participants were then organized in ascending order according to this number. The first individuals from the women’s and men’s lists will be invited for a face-to-face screening. If the randomly selected participant is ineligible for this study (i.e., failure to meet the inclusion criteria or withdrawal), he or she will be replaced by the next person on the list.

### Study protocol

After selection, the participants will undergo a 3-day baseline assessment at the Clinical Research Center of the Hospital de Clínicas de Porto Alegre, Brazil, including a clinical and nutritional interview, anthropometric and laboratory measurements, and questionnaires to assess mood, appetite, and food intake behavior. This same assessment will be performed at the end of the hypocaloric diet and tDCS intervention. The study timeline is shown in Fig. 1.

#### Clinical interview

The participants will undergo a standard evaluation that will include their medical history and a physical examination. Ethnicity will be classified as white or nonwhite according to official Brazilian methods of self-reported skin color [21]. Current smoking will be defined as active consumption in the last 3 months. Habitual alcohol consumption will be determined with a yes-or-no question. Sitting blood pressure will be measured in triplicate with a 1-minute interval between measurements after an initial cuff adjustment and 5-minute rest period (Omron BP785; OMRON Healthcare Co., Bannockburn, IL, USA). The mean of these measurements will be used to estimate systolic and diastolic arterial blood pressure. The clinical interview also will include the following:

- CAGE (cut down/annoyed/guilty/eye-opener) questionnaire screening to identify harmful alcoholic beverage consumption [22]
- Determination of socioeconomic status according to 2015 Brazilian economic classification criteria [23].
- Application of the Brazilian version of the International Physical Activity Questionnaire Short Form [24]. This questionnaire assesses three specific types of physical activity: walking, moderate-intensity activities, and vigorous activities. The intensity of total physical activity will be provided in (MET-min/wk; i.e., the sum of walking + moderate + vigorous MET-min/wk scores) [25].
- Habitual physical activity will be estimated in steps per day and will be assessed for 6 consecutive days with a digital pedometer (HJ-152-E; OMRON Healthcare Co., Kyoto, Japan) that will be provided to the participants [26].
- Application of the Brazilian version of the 36-item Short Form Health Survey (SF-36) to assess health-related quality of life [27]. This instrument is a well-validated measure of health status and health-related quality of life in which eight subscales are used to assess separate domains of health and related functioning, such as physical functioning, role physical, bodily pain, general health, vitality, social functioning, role emotional, and mental health. A single-item measure of comparative health is also included. The calculation procedures of the scores followed the recommendations of the SF-36 developers [28]. After the Likert-scaled items of each domain are summed, the SF-36 is scored from 0 (lowest health-related quality of life) to 100 (highest health-related quality of life).
- Application of the Epworth Sleepiness Scale, which provides a simple way of measuring general levels of sleepiness in ordinary life situations. In the validated Brazilian version, volunteers rate their chances of sleeping in situations on a scale of 0 to 3 [29].

#### Nutritional interview

The nutritional interview will be conducted at baseline and consists of a detailed investigation of dietary habits. Habitual food intake will be assessed using a 3-day weighed dietary records (WDR) technique (2 nonconsecutive weekdays and 1 weekend day) as previously standardized by our group [30]. The participants will receive a digital scale to weigh food (0–5 kg, Caumaq® EK3250; Cachoeira do Sul, Brazil) and a measuring cup for beverages (25–250 ml; Marinex®; Suzano, SP, Brazil). The foods should be registered on a form immediately after being measured. A trained registered dietitian will explain and demonstrate the proper use of the equipment to each subject. The baseline 3-day WDR will be performed the week before the dietary prescription.

#### Mood assessment

The Beck Depression Inventory, a 21-item self-report rating inventory, is probably the best known and most widely used depression scale. The Brazilian version of this tool will be applied at baseline and at the end of the study to measure characteristic attitudes and symptoms of depression. Scores range from 0 to 63, with 0, the minimal score, indicating no depression; 10 to 16 indicating mild to moderate depression; 17 to 29 indicating moderate to severe depression; and 30 to 36 indicating a state of severe depression [31, 32].

The State-Trait Anxiety Inventory (STAI), specifically the State Anxiety Scale (A-State), requires a description of how the participant feels at “this very moment” in relation to 20 items presented on a 4-point Likert scale (1 = not at all, 2 = somewhat, 3, = moderately, 4 = very much). This tool assesses the intensity of feelings of anxiety using items that measure subjective feelings of apprehension, tension, nervousness, worry, and activation/arousal of the autonomic nervous system. Participants will respond to the Brazilian version of the STAI at baseline and after the intervention. Scores range from 20 to 80 points, with higher values (> 49) indicating higher anxiety levels [33]. The participants will rate their attention and mood on a 100-mm visual analogue scale (VAS) word-anchored at each end (“terrible” through “very good”) by answering two questions: “How is your [attention/mood] at this very moment?”

#### Eating behavior assessment

*Hunger throughout the day* will be self-reported at baseline and at the end of the study (in the morning while the patient is still fasting) using a 100-mm VAS consisting of six word-anchored questions adapted from Haber et al. [34]. The participants will recall the intensity of their hunger upon waking, before lunch, 3 hours after lunch, before supper, 3 hours after supper, and before bedtime.

*Subjective satiety and hunger* scores will be assessed at baseline and at the end of the 4-week intervention using a 7-point VAS described by Holt and Miller [35]. On this scale, the participants will rate their hunger at “this very moment.” The answers range from − 3 = “extremely hungry” to 0 = “no particular feeling”, to + 3 = “extremely full”.

An additional 100-mm VAS, consisting of four word-anchored questions (“yes, very much” to “not at all”), will be administered before and after the 4-week intervention to assess the *desire to eat* something fatty, salty, sweet, or savory (as previously described) [36, 37].

The Food Craving Inventory–Brazilian version (FCI-Br), consisting of 23 food items distributed in 3 dimensions (sweet foods, high-fat foods, and traditional meals), will be applied at baseline and at the end of the study. The validated FCI-Br [38] has been adapted to include important characteristics of Brazilian eating habits. Like the original version [39], this tool begins with the question, “Over the past month, how often have you experienced a craving for the food listed below?” Each of the foods listed is evaluated using a 5-point Likert scale ranging from 1 = never to 5 = almost every day. The total score for each FCI dimension is calculated by the sum of the scores for each related item.

#### Anthropometric assessment

Anthropometric assessment will be performed with the participants barefoot and wearing light clothing. Height will be measured using a wall-mounted stadiometer and recorded to the nearest 0.1 cm. An InBody 230 tetrapolar body composition analyzer (InBody, Seoul, South Korea) will be used to measure body weight and composition via bioelectrical impedance. This procedure will be performed in the morning, with the participant fasting and having avoided exercise in the last 24 hours. BMI will be calculated as weight (kg)/height (m^2^). Mid-upper arm circumference will be measured at the midpoint of the upper right arm, between the acromion process and the tip of the olecranon. Waist circumference will be measured midway between the lowest rib margin and the iliac crest, near the umbilicus. Hip circumference will be measured at the maximal gluteal protrusion. Flexible, nonstretch fiberglass tape will be used for these measurements.

#### Metabolic assessment

Indirect calorimetry (MetaCheck; KORR Medical Technologies, Salt Lake City, UT, USA) will be used to determine resting metabolic rate (RMR). This measurement will be obtained at baseline and at the end of the study after a 12-hour fast and at least 24 hours without physical activity. The test will require a single 20-minute session, prior to which the participants will rest for at least 30 minutes. Indirect calorimetry analyzes the respiratory gases of the participants to measure their oxygen consumption and derives their RMR using Weir’s equation with assumed respiratory quotient = 0.83.

Adjusted resting metabolic rate (aRMR) will be defined using a strategy for RMR normalization that will involve multiple linear regression, a method that has been widely adopted in human obesity studies. The aim of normalizing RMR is to eliminate the influence of body size variation per se on RMR, such that the aRMR variable does not systematically vary with significant independent variables. Only then can RMR be compared across groups to determine the effect of an experimental intervention [40]. RMR is normalized by variables that, in multiple linear regression models, are shown to be independent determinants of RMR variation. To do this, the log_10_ of RMR is estimated as a linear function of log_10_ using regression, as recommended by Kaiyala and Schwartz [40].

#### Glycemic homeostasis and control

##### Assessment of glycemic control

Glycemic control will be assessed at baseline and at the end of the study, including a 75-g oral glucose tolerance test (OGTT) in which glucose will be measured at 0 and 120 minutes, glycated hemoglobin (A1c) and glycated albumin (GA) measurements, and a liquid meal tolerance test (LMTT) in which both glucose and insulin will be measured at − 30, − 15, 0, 30, 60, 120, 180, and 240 minutes.

For the LMTT, after a 12-hour fast, a flexible intravenous catheter will be placed in the antecubital space of the arm, and three blood samples (− 30, − 15, and 0 minutes) will be drawn over 15 minutes to determine the mean fasting values. A standard liquid formula meal will then be administered to all participants (400 ml Trophic 1.5, 600 kcal, 53% carbohydrates, 16% protein, 31% fat; Prodiet, Curitiba, Brazil), who will be instructed to consume it within 10 minutes. Additional blood samples will be collected at 30, 60, 120, 180, and 240 minutes after the meal. Serum and plasma will be divided into aliquots and immediately sent for analysis or frozen at − 80 °C for further analysis, as described below. Fasting glucose and insulin values will be calculated as the mean of the three basal values (− 30, − 15, and 0) from the LMTT. The AUC will be calculated for each LMTT using the trapezoidal rule [41]. Self-reported ratings of hunger and satiety and a self-reported rating of desire to eat will also be gathered immediately after each blood collection using the VAS described in the “Anthropometric assessment” section above.

GA is a laboratory test that reflects short-term glycemia due to the half-life of albumin, which is approximately 3 weeks. This test showed a better correlation with glucose excursions and postprandial hyperglycemia than A1c, which makes it more suitable for assessing short-term treatment efficacy [42].

##### Assessment of glycemic variability

Glycemic variability will be assessed using the FreeStyle® Libre™ Flash Glucose Monitoring System. This factory-calibrated interstitial glucose monitoring system uses a wired glucose oxidase enzyme coimmobilized on an electrochemical sensor that is worn on the back of the arm for up to 14 days [43]. Participants can obtain a real-time reading every minute by scanning the sensor with a reader. The data are transferred from the sensor to the reader memory and recorded automatically every 15 minutes. Glucose trends, rates, and direction for the past 8 hours are shown on the screen and can be uploaded to a computer for summary reports. The participants’ sensor data will be uploaded using FreeStyle® Libre™ version 1.0 software and will be saved into five files: one general file and one for each of the 4 weeks of treatment.

The mean amplitude of glycemic excursion (MAGE), which is the mean of blood glucose values exceeding 1 SD from the 24-hour mean blood glucose value, will be used as an index of glycemic variability [44]. To adapt MAGE for interstitial glucose, glucose values will be captured at 2-hour intervals from the minimum and maximum points of the glucose excursion plot. WebPlotDigitizer version 3.12 [45] will be used to calculate the minimum and maximum mean glucose values. This software accurately transforms a variety of plot types and images into numeric data.

A subgroup of individuals enrolled in this study will receive a FreeStyle® Libre™ unit to be used during the course of the 4-week intervention. Two sensors will be required to monitor each participant: The first will be placed on the first day of the intervention, 1 hour before the first tDCS session; the second will be placed after 14 days. A trained researcher will place the sensors on the participants according to the manufacturer’s instructions and will provide a brief explanation of their operation. For this trial, the participants will be instructed to check the sensor at least eight times daily: after waking up; just before and 2 hours after breakfast, lunch, and supper; and before sleeping.

#### Laboratory measurements

Twelve-hour fasting blood samples will be collected from all participants at baseline and at the end of the study to analyze lipid profile (total cholesterol, high-density lipoprotein [HDL] cholesterol, and triglycerides) and glycated hemoglobin (A1c), as well as to perform a 75-g OGTT in which glucose will be measured at 0 and 120 minutes. For the lipid profile, venous blood samples (5 ml) will be collected in separator gel serum tubes (Vacuette®; Greiner Bio-One, Monroe, NC, USA). Total cholesterol and triglycerides will be determined with an enzymatic colorimetric method (Cobas c702; Roche Diagnostics, Indianapolis, IN, USA). HDL cholesterol will be determined with a homogeneous enzymatic colorimetric assay (Cobas c702). For A1c, a venous blood sample (4 ml) will be collected in ethylenediaminetetraacetic acid (EDTA)-treated tubes (Vacuette®), and levels will be determined with an high-performance liquid chromatography method (VARIANT II TURBO HbA1c kit; Bio-Rad Laboratories, Hercules, CA, USA) certified by the National Glycohemoglobin Standardization Program, which is aligned with that of the International Federation of Clinical Chemistry. The clinical pathology department of the Hospital de Clínicas de Porto Alegre participates in the A1c External Quality Assurance Program, in which it has demonstrated adequate performance [46]. For plasma glucose from OGTT, venous blood samples (4 ml) will be collected in Vacuette® tubes with sodium fluoride, and results will be determined with an enzymatic ultraviolet (UV)-hexokinase method (Cobas c702).

On a different day from the OGTT, an LMTT will be conducted at baseline and at the end of the study to assess not only glucose and insulin response to a meal but also changes in C-peptide, to estimate insulin sensitivity and β-cell function, and to evaluate changes in hormones such as PYY, GLP-1, PP, and ghrelin, which are involved in satiety and hunger regulation [47]. To assess this eight-point glucose-insulin curve (− 30, − 15, 0, 30, 60, 120, 180, and 240 minutes), blood samples will be collected into Vacuette® tubes with sodium fluoride for glucose (4 ml) and with separator gel serum tubes for insulin (5 ml). Serum and plasma will be obtained after centrifugation (3500 rpm, 15 minutes, room temperature) and kept on ice until analysis. The measurements will be taken at the Hospital de Clínicas de Porto Alegre Clinical Pathology Unit. Glucose will be determined using an enzymatic UV-hexokinase method (Cobas c702), whereas insulin will be determined using a chemiluminescence microparticle immunoassay (ARCHITECT Ci4100; Abbott Diagnostics, Lake Forest, IL, USA).

For further hormonal analysis, two venous blood samples (4 ml) will be collected in EDTA-treated tubes (Vacuette®) during the LMTT. In one of the tubes, 40 μl of dipeptidyl peptidase 4 inhibitor (K579; Tocris/Bio-Techne, Minneapolis, MN, USA) will be immediately added to prevent degradation; plasma will be obtained by centrifugation at 1500 rpm and 4 °C for 10 minutes, with the aliquots stored at − 80 °C. The other plasma sample will be centrifuged at 3500 rpm and room temperature for 15 minutes, aliquoted, and stored at − 80 °C for further analysis. PYY_3–36_ is planned to be measured using a commercially available enzyme-linked immunosorbent assay kit (catalogue no. EKE-059-02; Phoenix Pharmaceuticals, Burlingame, CA, USA) according to the manufacturer’s instructions. Fasting and postprandial plasma ghrelin, active GLP-1, C-peptide, and PP concentrations are planned to be measured in the same assay using the human gut hormone panel LINCOplex kit (EMD Millipore, Burlington, MA, USA). This multiplex assay kit uses antibody-immobilized beads to simultaneously quantify these peptide hormones. These analyses are planned to be done in the end of the study when the last individual completes the protocol.

For GA, fasting venous blood samples (5 ml) will be collected during the LMTT in separator gel serum tubes (Vacuette®). Serum will be obtained after centrifugation (3500 rpm, 15 minutes, room temperature) and stored at − 80 °C for subsequent determination. GA measurement will involve the GlycoGap enzymatic method (Diazyme Laboratories, Poway, CA, USA), which measures the amount of glycated serum protein (mmol/L) but not GA specifically. However, due to similar reaction principles, GA levels can be calculated from the total albumin concentration. This method had more analytical precision, with intra- and interassay coefficients of variation of 3.5% and 8.7%, respectively, in previous analyses performed in our laboratory [48].

#### Assessment of insulin sensitivity and β-cell function

Insulin sensitivity and β-cell function will be estimated using static and dynamic measures based on glucose, C-peptide, and insulin measurements taken during the LMTT. The homeostatic model assessment (HOMA) insulin resistance (HOMA-IR) index can be calculated in two ways: estimating IR, in which HOMA-IR = fasting plasma insulin (FPI; μUI/ml) × fasting plasma glucose (FPG; mmol/L)/22.5, and estimating β-cell function, in which HOMA-β = (20 × FPI)/(FPG − 3.5) [49]. The calculator used for this purpose is available online [50].

The LMTT provides a means of assessing insulin secretion patterns in more physiological conditions because these tests include the incretin effect, which follows oral nutrient ingestion. Insulin sensitivity and β-cell responsivity to glucose will be determined as previously described [51]. Plasma glucose and insulin concentrations will be used to determine LMTT indices of insulin sensitivity (Matsuda insulin sensitivity index [MISI]). Pancreatic β-cell function will be determined by calculating the LMTT disposition index.

#### Intervention procedures

At the end of the baseline evaluation, each participant will be prescribed a hypocaloric diet aimed at reducing his or her initial weight by at least 3% in 4 weeks. While on the diet, the participants will receive five weekly visits for tDCS intervention, either active or sham according to prior randomization. A registered dietitian blinded to the tDCS intervention will visit each participant weekly to confirm diet adherence and to make any necessary adjustments to the diet. Participants will be strongly instructed not to change their current physical activity during the trial. Body weight will also be assessed weekly with the same bioelectrical impedance scale used in the baseline evaluation (3-hour fast, light clothes, barefoot).

If a participant withdraws or is removed from the study for any reason, all research-related activities for that individual will be immediately discontinued. Final clinical, anthropometric, and laboratory examinations will be collected from willing participants. Intention-to-treat and per-protocol analyses will be performed.

##### Hypocaloric diet

The energy deficit will be calculated for each participant individually using the online Body Weight Planner program, which is a validated dynamic simulation model of human metabolism recommended by the National Institute of Diabetes and Digestive and Kidney Diseases for prescribing weight loss diets [52]. Using the information on age, sex, height, weight, and physical activity level, the number of kilocalories per day needed to alter a specific amount of weight in a certain number of days can be determined.

The prescribed hypocaloric diets will conform to American Diabetes Association specifications for overweight or obese patients with type 2 diabetes or prediabetes. The planned macronutrient profile will be 45–50% of total energy intake as carbohydrates, with emphasis on a low glycemic index; 15–25% as protein; ≤ 30% from fat, with unsaturated fats being the main source (saturated fat limited to < 7%); and ≥ 20 g/d or 14 g/1000 kcal/d as fiber [53].

Compliance with this diet and changes in food consumption from baseline will be assessed using the 3-day WDR technique at three different times: at baseline, as described in the “Eating behavior assessment” section above, at the end of the first 2 weeks, and again during the last 2 weeks of the study, totaling nine WDRs. The diet composition, obtained from the prescribed diet and from the 3-day WDRs, will be analyzed (U.S. Department of Agriculture table [54]) in NutriBase 17 Pro Edition software (version 17.2; CyberSoft, Inc., Phoenix, AZ, USA). The mean values for energy and nutrients consumed during the 3-day WDR will be calculated. Patients will be classified as adherent or nonadherent according to whether or not they will reach their calculated energy goals and diet recommendations.

##### Transcranial direct current stimulation

The tDCS technique consists of applying a weak constant electrical current (typically 1.0 to 2.0 mA) through two surface electrodes placed on the scalp to modulate brain activity in a polarity-dependent fashion; that is, anodal stimulation typically increases spontaneous neuronal excitation (depolarization), whereas cathodal stimulation has the opposite effect (hyperpolarization).

A battery-driven direct current stimulator called the Chattanooga Ionto™ Dual Channel Electrophoresis System (DJO Global, Guildford, UK) will be used in this protocol. Direct current (2 mA) will be delivered over the right DLPFC through a pair of 35-mm^2^, 0.9% saline-soaked surface sponge electrodes placed according to the 10–20 International System of Electrode Placement (EEG 10/20). The right DLPFC position will be found according to Beam et al.’s methods [55]. This system accounts for variability in subject skull size by using certain percentages of the circumference and distances between four basic anatomic landmarks (right and left tragus, nasion, and inion). We will use Beam F3 Locator software, which was developed to more accurately determine where the electrodes should be placed on the skull [56].

The participants will be randomized to receive either active or sham tDCS treatment. While following the hypocaloric diet, they will undergo 20-minute daily tDCS sessions each weekday (including holidays) for 4 weeks (20 total sessions). If a scheduled tDCS session will be missed for any reason, it will be made up the following weekend. The participants will be instructed not to consume food 3 hours before each stimulation session. Each session will be conducted at the same time of day for each participant, and only trained technicians will perform tDCS.

For active tDCS, the electrodes will be placed with the anode electrode over the right DLPFC (F4) and the cathode electrode over the left DLPFC (F3), using the technique described above. The current will be ramped up for 15 seconds until it reaches 2 mA, and the participants will then be stimulated for 20 minutes. The current will then be ramped down for another 15 seconds and will be turned off. For sham tDCS, the electrodes will be placed identically to those in the active group, but the current will be ramped up to 2 mA and then back down in the first and last 30 seconds of the 20-minute session. As a result, the participants will feel the initial itching sensation associated with turning on the device but will receive no stimulation for 19 minutes of the session.

During the sessions, the subjects will also be exposed to a series of high-calorie food images previously demonstrated to stimulate the appetite. Three different sets of 200 pictures of high-calorie and highly palatable foods, such as sweets, high-fat foods, and traditional meals, will be shown to participants as a video presentation during the 20-minute sessions, with a 6-second interval between pictures. The participants will be asked to pay attention to these images and turn off phones, pagers, or other devices that might distract their attention.

In addition, the participants’ subjective feelings of desire to eat, mood, and attention will be assessed immediately before and after the first, tenth, and twentieth tDCS sessions with a VAS, as described in the “Anthropometric assessment” section above. The aim is to verify the presence of acute effects on the desire to eat after visual stimuli associated with tDCS.

Because effects such as headache, scalp burning, tingling, and dizziness have previously been associated with tDCS, the participants will be asked about adverse effects twice each week at the end of a stimulation session, as well as whether they had experienced any such effects after the previous session.

### Outcomes

The primary outcomes will be reductions in body weight and BMI from baseline and reduction or maintenance of the prescribed energy and macronutrient intake during the 4-week intervention. The secondary outcomes will be reduced hunger, desire to eat, and food craving scores compared with baseline; reductions in fasting and postprandial responses of plasma glucose, insulin, GA, hunger hormones such as ghrelin; and an increase in the blood levels of hormones that participate in the regulation of satiety, such as PYY_3–36,_ GLP-1, and PP [47]. These hormones will be measured to understand mechanisms behind a possible effect of tDCS in weight, desire to eat, food cravings, or glycemic control and variability.

The exploratory outcomes will include the number of participants achieving a 3% reduction or more in body weight compared with baseline; improvements in insulin sensitivity and β-cell function compared with baseline; changes in body composition, such as reduced waist and hip circumferences and reduced body fat mass, compared with baseline; changes in glycemic variability, such as reduced MAGE compared with baseline; changes in depression and anxiety scores compared with baseline; and acute changes in subjective feelings of desire to eat, mood, and attention between pre- and post-tDCS sessions.

### Statistical procedures

#### Sample size

The sample size was calculated on the basis of a significant reduction in the amount of calories ingested after a single 20-minute session of active tDCS (*F*_1,8_ = 8.4, *p* = 0.02, ηp^2^ = 0.5) [12]. Three types of analysis were performed using G*Power 3.1.9.2® software, considering a power of 80% and an α of 0.05, as follows: effect size (*f*) = 1.0, 10 subjects; effect size (*f*) = 0.8, 12 subjects; and effect size (*f*) = 0.6, 20 subjects. In another study, a 14.2% reduction in the amount of calories ingested was observed after 8 consecutive days of active tDCS sessions. The authors considered a medium effect size (*d*) > 1.09 as sufficient for their findings [13]. Following these assumptions, a total of at least ten subjects would be required in each group to detect a medium-sized effect. Because of possible dropouts, we will include an additional 40% of participants in this study.

#### Randomization

The block randomization method is designed to randomize subjects into groups that result in equal sample sizes. In the present study, two blocks of six participants and four blocks of four participants will be used, stratified by sex (three female and three male blocks) in a 1:1 ratio to receive one of two interventions: daily sessions of active tDCS associated with hypocaloric diet (active group) or daily sessions of sham tDCS associated with hypocaloric diet (sham group). Each block will consist of opaque, sealed envelopes, identified as female or male, which will contain sealed tickets labeled “Diet + active tDCS” or “Diet + sham tDCS” in a 1:1 ratio. Only one ticket will be drawn for each volunteer, which will occur near the beginning of the study, after the hypocaloric diet has been prescribed. The participants, as well as all other investigators except those who applied tDCS (i.e., the investigators who performed the screening and clinical assessment visits and those who prescribed the hypocaloric diet), will be blinded to the different interventions. Randomization (DAN), patient selection, dietary intervention (CdA, RCF), and tDCS sessions (DAN) will be performed by different researchers to ensure study blindness. The raters (CdA, RCF, FG) neither administered nor will be present during tDCS sessions.

#### Data analysis

To compare the participants’ baseline characteristics, Student’s *t* test and the chi-square test will be used as appropriate. The results will be expressed as mean ± SD, mean (95% CIs), or the number of patients with the characteristic (percent). The Shapiro-Wilk test will be used to verify data normality; variables with non-Gaussian distribution, such as insulin and the HOMA index, will be logarithmically transformed before analysis when necessary. The aRMR will be performed using logarithmically transformed data, as recommended by Kaiyala and Schwartz [40].

The effects of tDCS between groups over time will be analyzed using the generalized estimating equation (GEE), including tDCS group, time, and the group- and subgroup-by-time interaction as predictors. All GEE models will be followed by a Bonferroni post hoc analysis and the least significant difference test to identify significant differences detected by the GEE. Gamma distribution with log link function will be used for variables with non-Gaussian distribution. After treatment of the first ten participants, an interim security analysis was planned, and the trial may be stopped prematurely because of safety concerns, such as severe psychiatric and neurologic reactions. The outcomes will be presented as *p* values for the included factors and estimated marginal means, with corresponding 95% CIs for each tDCS group at each time point. A *p* value < 0.05 will be considered statistically significant. IBM SPSS Statistics version 19.0 statistical software (IBM, Armonk, NY, USA) will be used for the analyses.

## Discussion

To our knowledge, this is the first study to investigate whether sessions of repetitive tDCS over the right DLPFC may add additional benefits to weight loss in subjects undergoing a calorie-restricted diet. The evidence-based treatment options for overweight and obesity control currently available are lifestyle intervention, pharmacotherapy, and bariatric surgery [4]. Lifestyle interventions are the first option for weight loss, given their low cost and minimal risk of adverse effects and complications. Bariatric surgery is frequently recommended in accordance with strict treatment criteria for patients who do not respond sufficiently to lifestyle therapy and/or pharmacotherapy [4]. Thus, the use of tDCS, a well-tolerated, safe, and noninvasive technique, would act as a complementary treatment to lifestyle therapy for managing overweight and obesity.

The positioning of the electrodes is considered an important determinant of stimulation effectiveness. For this protocol, the choice of anodal stimulation on the right DLPFC and cathodal stimulation on the left DLPFC (anodal right/cathodal left [AR/CL]) was based on previous studies that found a significant effect in reducing food intake and desire to eat associated with this position, in both healthy [11, 12] and overweight or obese individuals [10]. To date, most studies reporting a reduction in food craving have used the AR/CL electrode positioning over the DLPFC, which presumably increases activity in the right DLPFC (F4 position) and inhibits activity in the left DLPFC (F3 position) [10–12, 17, 57].

This protocol was designed to include a total of 20 tDCS sessions on 20 consecutive weekdays, each lasting 20 minutes. The effects of a single tDCS session have been assessed in several studies, showing significant reductions in the desire to eat and food consumption [10–12, 17, 57, 58], with the exception of two studies [59, 60]. The use of 20 repetitive tDCS sessions has not yet been reported in the literature for this purpose. However, a European group of experts recently reported that daily tDCS sessions (between 5 and 30 sessions) have been commonly used for other clinical conditions, such as chronic pain, stroke, schizophrenia, substance abuse, and other addictions [61]. Only one study so far has found a reduction in the desire to eat after 5 days of repetitive tDCS over the right DLPFC in overweight and obese individuals [18]. Long-term effect studies in which the cumulative effects of tDCS can be analyzed are still sparse. This study was also motivated by evidence that the magnitude of the behavior change might be associated with the number of sessions needed to observe a change in eating habits [62].

Although daily contact between the research team and participants could positively influence weight loss and adherence to the hypocaloric diet, as well as improve depression and anxiety, randomization to either real or sham tDCS treatment should mitigate this potential bias.

This study may provide important data on tDCS as a potential complementary treatment for hypocaloric diet in overweight and obesity management and as an alternative to medications or bariatric surgery. Authors of a recent meta-analysis concluded that noninvasive brain stimulation has significant effects on food craving [63]. Because eating behavior is an important component that can enhance adherence to prescribed diets, we believe that the potential of tDCS to modulate eating behavior could contribute to better adherence to dietary treatment and thus to weight loss and better quality of life.

### Trial status

This trial is currently ongoing. RCT recruitment started in March 2016.

## Additional file

Additional file 1: SPIRIT 2013 checklist: recommended items to address in a clinical trial protocol and related documents. (DOCX 49 kb)

## Abbreviations

AR/CL: Anodal right/cathodal left
aRMR: Adjusted resting metabolic rate
BMI: Body mass index
CKD-EPI: Chronic Kidney Disease Epidemiology Collaboration
DLPFC: Dorsolateral prefrontal cortex
EDTA: Ethylenediaminetetraacetic acid
FCI-Br: Food Craving Inventory–Brazilian version
FPG: Fasting plasma glucose
FPI: Fasting plasma insulin
GA: Glycated albumin
GEE: Generalized estimating equation
HDL: High-density lipoprotein
HOMA: Homeostatic model assessment
HOMA-IR: Homeostatic model assessment of insulin resistance
LMTT: Liquid meal tolerance test
MAGE: Mean amplitude of glycemic excursion
OGTT: Oral glucose tolerance test
RMR: Resting metabolic rate
SF-36: 36-Item Short Form Health Survey
STAI: State-Trait Anxiety Inventory
tDCS: Transcranial direct current stimulation
TSH: Thyroid-stimulating hormone
UV: Ultraviolet
VAS: Visual analogue scale
WDR: Weighed dietary records
